# Knowledge of lactose intolerance in Italian adults: a cross‑sectional assessment

**DOI:** 10.1007/s00394-026-04106-4

**Published:** 2026-09-24

**Authors:** Giulia Carioni, Simona Dominici, Monica Guglielmetti, Angela Zinnai, Filippo Fassio, Michele Di Stefano, Patrick Maisonneuve, Maria Sole Facioni, Patrizia Gnagnarella

**Affiliations:** 1 Division of Epidemiology and Biostatistics, IEO European Institute of Oncology IRCCS, 20141 Milan, Italy; 2 Department of Medicine, University of Udine, 33100 Udine, Italy; 3 AILI-Italian lactose-intolerant patients’ association Aps, 55100 Lucca, Italy; 4 ELLEFREE S.R.L, Polo Tecnologico Lucchese, 55100 Lucca, Italy; 5 Human Nutrition Center, Department of Public Health, Experimental and Forensics Medicine, University of Pavia, 27100 Pavia, Italy; 6 Department of Agriculture, Food and Environment, University of Pisa, 56124 Pisa, Italy; 7 SOSD Allergology and Clinical Immunology, Azienda USL Toscana Centro, San Giovanni di Dio Hospital, 50143 Florence, Italy; 8 Department of Internal Medicine, IRCCS S. Matteo Hospital Foundation, 27100 Pavia, Italy; 9 Department of Clinical Medicine and Surgery, University of Naples “Federico II”, 80131 Naples, Italy

**Keywords:** Lactose intolerance, Nutrition knowledge, Dietary management, Diagnostic accuracy, Health education, Food intolerance beliefs

## Abstract

**Purpose:**

To assess knowledge of lactose intolerance (LI) in Italian adults, identifying major knowledge gaps and factors associated with awareness.

**Methods:**

A cross-sectional study was conducted between December 2024 and March 2025 among Italian adults (≥ 18 years). A newly developed 39-item online survey assessed knowledge of LI across four modules (definition, symptoms, diagnosis, and management). The knowledge score (range: − 39; +39) was calculated and participants were classified into three knowledge levels based on the tertile distribution of scores: “low knowledge”, “intermediate knowledge”, and “high knowledge”’. Univariable and multivariable ordinal logistic regression were carried out to investigate the association between respondents’ characteristics and LI knowledge.

**Results:**

A total of 2025 respondents (85.4% female) were included in the analysis. The mean knowledge score was 15.4 ± 7.6. Overall, 36.6% of respondents showed low, 32.2% intermediate, and 31.2% high knowledge. Common misconceptions concern the belief that trace amounts of lactose (e.g., in lactose-free products or medications) can trigger symptoms and that a lactose-free diet does not pose a risk for nutritional deficiencies. Questions on unvalidated diagnostic methods elicited many “I don’t know” responses, reflecting uncertainty about their reliability. Female gender, confirmed LI diagnosis, higher education and nutrition training were associated with higher knowledge, with the latter showing the strongest association (Odds Ratio 10.8, 95% CI: 8.30–14.2).

**Conclusion:**

These findings highlight persistent misconceptions about LI and underscore the need for targeted educational strategies to improve public understanding and reduce inappropriate diagnostic and dietary practices.

**Supplementary Information:**

The online version contains supplementary material available at https://doi.org/10.1007/s00394-026-04106-4.

## Introduction

Lactose is the main carbohydrate in milk and dairy products and is also widely used as a bulking agent in food processing and pharmaceutical formulations [1, 2]. Upon ingestion, lactose is hydrolyzed in the duodenum by lactase-phlorizin hydrolase (LPH), an enzyme located on the brush border of enterocytes. Like other disaccharides, lactose must be split into its monosaccharide components, glucose and galactose, before absorption [3]. Lactase deficiency or decreased enzymatic activity impairs lactose digestion leaving undigested lactose in the intestinal lumen, where it undergoes fermentation by the gut microbiota. The resulting osmotic water retention and production of gases and short-chain fatty acids contribute to the pathophysiology and symptoms of lactose intolerance [4].

Lactose intolerance (LI) is defined as the occurrence of gastrointestinal symptoms following lactose ingestion in individuals with lactose malabsorption due to lactase deficiency. LI can be classified into four forms: congenital, primary, secondary, and developmental. Symptoms commonly include cramp-like abdominal pain, bloating, flatulence, gastric heaviness, a sensation of fullness, diarrhea, constipation, or alternating bowel habits, although extraintestinal manifestations have also been reported [5]. Symptom severity varies considerably among individuals and is influenced by several factors, including lactose dose, individual tolerance threshold, and dietary context. Diagnosis is primarily based on the Hydrogen Breath Test (H₂-LBT), currently considered the gold standard, or alternatively on the more recent 13 C-Lactose Breath Test (13 C-LBT) [6–8]. Genetic testing for the *LCT C/T − 13,910* polymorphism can also be used to identify primary lactase deficiency [9, 10]. However, because LI symptoms overlap with those of other gastrointestinal disorders, diagnosis may often be delayed [11–13]. Furthermore, inadequate post-diagnostic support and misinformation may also lead to unwarranted dietary restrictions, impairing nutritional status and quality of life [12–15]. LI is highly prevalent worldwide, although its distribution varies considerably across geographical regions because of differences in lactase persistence. Approximately 65–70% of the global adult population is estimated to have reduced ability to digest lactose. In Europe, prevalence ranges from about 5–10% in Northern countries to 70% in Southern Europe [3, 16–18]. In Italy, lactase non-persistence is estimated to affect approximately 50% of the population, with a north-to-south gradient [18].

Despite its high prevalence, substantial public confusion persists regarding LI. Misinterpretation of symptoms often leads individuals to self-diagnose or rely on non-validated testing methods [19–21]. Consequently, dairy products may be unnecessarily restricted or completely excluded without proper professional guidance, potentially compromising nutritional adequacy, particularly regarding calcium, vitamin D, and protein intake [12, 20, 22]. Conversely, limited awareness about hidden sources of lactose may result in unintentional exposure and persistent symptoms. Accurate diagnosis and appropriate information are therefore essential to support individualized dietary management while avoiding unnecessary restrictions.

Assessing public understanding of LI is crucial for identifying misperceptions and areas requiring targeted education. However, despite the extensive literature on LI, only a few studies have specifically investigated the knowledge of the condition among the general population [23, 24]. To address this gap, an inter-society working group among the Italian Society of Human Nutrition (SINU), the Italian Lactose-Intolerant Patients’ Association (AILI) and members of the Food Composition Database for Epidemiological Studies in Italy (BDA) was specifically established. The group includes experts in nutrition, food composition, food technology and medicine, providing a multidisciplinary approach.

The present study aimed to assess knowledge of LI among Italian adults, identifying the current gaps in information and misconceptions that may often lead to inappropriate diagnosis and dietary management.

## Method

### Study design and participants

A cross-sectional study was conducted to assess knowledge of clinical, pathophysiological, diagnostic and therapeutic aspects of LI. The following inclusion criteria were applied: participants of any gender, aged ≥ 18 years and living in Italy were eligible for recruitment between December 2024 and March 2025. Adults unable to provide consent were ineligible to participate. Enrollment was based on voluntary participation. The survey was disseminated through newsletters and the social media channels of the associations and institutes involved in the project. Additionally, scientific and professional nutrition societies were asked to disseminate the survey among their contacts, employees or members. Word of mouth and leaflets were used to further expand the reach of participants.

The study was approved by the Lombardy Ethics Committee 2 (L2-201 of 25/9/2024) and was conducted according to the Declaration of Helsinki principles. Informed consent was obtained from all participants before the study initiation.

### Survey

The present survey was newly developed by conducting an exploratory review of the literature related to LI, with particular focus on dietary recommendations, and existing related questionnaires [25, 26]. As no questionnaire specifically assessing knowledge of LI was identified, a new survey was developed using existing related questionnaires as methodological references. The development process is reported in Supplementary Fig. 1. Four main areas were identified to assess knowledge (definition, symptoms, diagnosis and management). An initial pool of 47 items was generated and evaluated by the working group to ensure content and face validity [27, 28]. A pre-pilot test with university students and a pilot study involving hospital employees were conducted to assess item clarity and feasibility. Based on the feedback received, modifications and refinements were made to enhance the understanding and optimize the sequence of questions.

The final survey consisted of 52 questions. The demographics comprised 13 items, collecting personal information (e.g., age, gender, educational level, nutrition-related qualifications, occupation), as well as self-reported LI status. The knowledge section (see Supplementary Table 1) consisted of 39 items, divided into four thematic modules: (i) definition of lactose, dietary sources, and causes of LI (8 items); (ii) common symptoms (12 items); (iii) diagnosis (9 items); and (iv) management of LI, including available medications and supplements (10 items). Both multiple choice and true/false questions were used to give a broader overview of the level of knowledge. An “I don’t know” response option was included for each item, to minimize random responses. All 39 knowledge items contributed equally to the overall knowledge score.

The survey was developed using Microsoft Forms. The ‘one response per person’ setting was used, in order to prevent duplicate survey submissions. All survey questions were set as mandatory, preventing partial completions. All data were collected anonymously.

### Knowledge score

Based on responses to the 39 specific knowledge items, we computed a knowledge score as the sum of correct and incorrect answers. A three-level scoring system was adopted: +1 point was assigned for each correct answer, 0 points for “I don’t know” responses, and − 1 point for each incorrect answer. All items were equally weighted, resulting in a total knowledge score ranging from − 39 to + 39. Participants were then categorized into three relative knowledge levels based on the tertile distribution of scores: those with scores ≤ 12 were categorized as having ‘low knowledge’; those scoring between 13 and 19 as having ‘intermediate knowledge’; those with scores ≥ 20 as having ‘high knowledge’. A quality check was performed: all responses were carefully reviewed to identify inconsistencies and the time taken to complete the survey was monitored. Participants with unreliable responses (e.g., inconsistent demographic information such as a 20-year-old respondent reporting being widowed and retired) or implausibly short completion time (e.g., less than 2 min, suggesting insufficient time to read questions) were excluded from the final analysis.

### Statistical analysis

The online survey aimed to reach a representative sample of Italian adults. Based on the estimated prevalence of LI in the Italian population (approximately 50%) [18, 29], the required sample size was set at 385 individuals with a precision of 0.05 at a 95% confidence level, to ensure a sufficient number of participants with LI (https://it.surveymonkey.com/learn/research-and-analysis/sample-size-calculator/*).* Considering the interest in the topic and the number of individuals reached by the mailing lists of the associations involved (around 10,000), and anticipating a response rate of 27% [30], we hypothesized to reach about 1500 participants.

Participants’ characteristics and their responses to questions related to LI knowledge were presented as numbers and frequencies using contingency tables. Mean and standard deviation (SD) were calculated for continuous variables and proportions and percentages for categorical ones. The chi-square test for categorical variables and the Student’s t-test for continuous variables were used to assess differences between groups. Univariable and multivariable ordinal logistic regression assuming proportional odds were used to confirm the association between respondents’ characteristics and overall knowledge about LI. The proportional odds assumption was formally tested and implies that the effect of a co-variable on overall knowledge score is proportional across all the categories and that the odds ratios are the same for all the comparisons (‘intermediate knowledge’ versus ‘low’, or ‘high Knowledge’ versus ‘intermediate’). Analyses were performed with the SAS software version 9.4 (Cary, NC, USA). A *p*value < 0.05 was set for statistical significance.

## Results

### Sample characteristics

A total of 2038 responses were collected between December 2024 and March 2025. After a data quality check, 2025 responses were included in the final analysis: one respondent was excluded due to an implausible completion time and 12 due to inconsistencies in their answers within the personal information section. The characteristics of the study population are presented in Table 1. Most respondents were female (85.4%), aged between 35 and 64 years (58.5%), from the north-west of Italy (39.6%), graduated (66.2%), employed (76.1%) and single (46.9%). Participants were classified into three groups based on their self-reported LI status: ‘confirmed’ intolerant, if they reported LI supported by validated tests (lactose breath test, LCT-13910 genetic test, or both); ‘suspected’ of having LI, if they reported a reduction in symptoms following the exclusion of lactose from their diet without the support of validated tests [31], or they performed a non-validated test (e.g. food intolerance tests, Cytotest, Vega test, DRIA test) [32–34]; and ‘tolerant’ if they did not report any clinical manifestations relating to lactose intake. Overall, 35.1% (711) were considered intolerant, 19.8% (401) suspected intolerant, while 45.1% (913) reported not having the condition. Finally, 23.1% (467) of respondents held a degree in nutrition-related disciplines, of whom 87.8% were female. Regional differences were observed: most respondents from the South of Italy (47.8%) and islands (44.1%) were lactose intolerant, whereas respondents from North were lactose tolerant (51.8% in the north-west; 42.1% in the north-east). Lactose-intolerant individuals were mainly women (91%), aged 35–64 years (62.7%), without a university degree (47%), employed (72%) and not experts in nutrition (94.7%) (data not shown in table).

**Table 1:** Respondents’ baseline characteristics for the total sample (*n* = 2025) and divided by presence/absence/suspicion of lactose intolerance, nutrition knowledge (experts/inexperts) and sex

	Total	Lactose tolerant	Suspect intolerant	Lactose intolerant	Inexpert	Nutrition expert	Male	Female
	*N* = 2025	*N* = 913	*N* = 401	*N* = 711	*N* = 1558	*N* = 467	*N* = 284	*N* = 1730
	N (col %)	N (row %)	N (row %)	N (row %)	N (row %)	N (row %)	N (row %)	N (row %)
*Gender* ^*$*^								
Male	284 (14.0)	169 (59.5)	52 (18.3)	63 (22.2)	229 (80.6)	55 (19.4)	–	–
Female	1730 (85.4)	738 (42.7)	345 (19.9)	647 (37.4)	1320 (76.3)	410 (23.7)	–	–
*Age*								
18–34	750 (37.0)	379 (50.5)	141 (18.8)	230 (30.7)	508 (67.7)	242 (32.3)	140 (18.7)	608 (81.1)
35–64	1185 (58.5)	499 (42.1)	240 (20.3)	446 (37.6)	974 (82.2)	211 (17.8)	127 (10.7)	1050 (88.6)
65+	90 ( 4.4)	35 (38.9)	20 (22.2)	35 (38.9)	76 (84.4)	14 (15.6)	17 (18.9)	72 (80.0)
*Italian region*								
North-West	801 (39.6)	415 (51.8)	174 (21.7)	212 (26.5)	690 (86.1)	111 (13.9)	138 (17.2)	659 (82.3)
North-East	280 (13.8)	118 (42.1)	67 (23.9)	95 (33.9)	177 (63.2)	103 (36.8)	35 (12.5)	243 (86.8)
Central	481 (23.8)	213 (44.3)	80 (16.6)	188 (39.1)	318 (66.1)	163 (33.9)	55 (11.4)	422 (87.7)
South	320 (15.8)	119 (37.2)	48 (15.0)	153 (47.8)	248 (77.5)	72 (22.5)	40 (12.5)	279 (87.2)
Islands	143 ( 7.1)	48 (33.6)	32 (22.4)	63 (44.1)	125 (87.4)	18 (12.6)	16 (11.2)	127 (88.8)
*Education level*								
High school or less	685 (33.8)	201 (29.3)	150 (21.9)	334 (48.8)	682 (99.6)	3 ( 0.4)	94 (13.7)	587 (85.7)
Bachelor’s degree	450 (22.2)	235 (52.2)	92 (20.4)	123 (27.3)	285 (63.3)	165 (36.7)	71 (15.8)	379 (84.2)
Master’s degree	698 (34.5)	371 (53.2)	120 (17.2)	207 (29.7)	443 (63.5)	255 (36.5)	87 (12.5)	605 (86.7)
Post-graduate /PhD	192 ( 9.5)	106 (55.2)	39 (20.3)	47 (24.5)	148 (77.1)	44 (22.9)	32 (16.7)	159 (82.8)
*Employment status*								
Student	216 (10.7)	113 (52.3)	53 (24.5)	50 (23.1)	173 (80.1)	43 (19.9)	46 (21.3)	169 (78.2)
Employed	1541 (76.1)	730 (47.4)	299 (19.4)	512 (33.2)	1139 (73.9)	402 (26.1)	211 (13.7)	1324 (85.9)
Housewife	96 ( 4.7)	18 (18.8)	16 (16.7)	62 (64.6)	95 (99.0)	1 ( 1.0)	0 ( 0.0)	95 (99.0)
Retiree	92 ( 4.5)	31 (33.7)	22 (23.9)	39 (42.4)	85 (92.4)	7 ( 7.6)	16 (17.4)	75 (81.5)
Unemployed	80 ( 4.0)	21 (26.3)	11 (13.8)	48 (60.0)	66 (82.5)	14 (17.5)	11 (13.8)	67 (83.8)
*Marital status*								
Married	932 (46.0)	420 (45.1)	176 (18.9)	336 (36.1)	741 (79.5)	191 (20.5)	109 (11.7)	819 (87.9)
Widow(er)	22 ( 1.1)	9 (40.9)	4 (18.2)	9 (40.9)	18 (81.8)	4 (18.2)	2 ( 9.1)	20 (90.9)
Divorced	121 ( 6.0)	49 (40.5)	29 (24.0)	43 (35.5)	103 (85.1)	18 (14.9)	11 ( 9.1)	108 (89.3)
Single	950 (46.9)	435 (45.8)	192 (20.2)	323 (34.0)	696 (73.3)	254 (26.7)	162 (17.1)	783 (82.4)
*Lactose intolerance*								
No	913 (45.1)	–	–	–	544 (59.6)	369 (40.4)	169 (18.5)	738 (80.8)
Suspected	401 (19.8)	–	–	–	341 (85.0)	60 (15.0)	52 (13.0)	345 (86.0)
Confirmed	711 (35.1)	–	–	–	673 (94.7)	38 ( 5.3)	63 ( 8.9)	647 (91.0)
*Nutrition expert*								
No°	1558 (76.9)	544 (34.9)	341 (21.9)	673 (43.2)	–	–	229 (14.7)	1320 (84.7)
Yes	467 (23.1)	369 (79.0)	60 (12.8)	38 ( 8.1)	–	–	55 (11.8)	410 (87.8)

^$^11 responders preferred not to specify their gender

°Include 550 responders interested in nutrition topics

### Knowledge about lactose intolerance

**Fig. 1:**
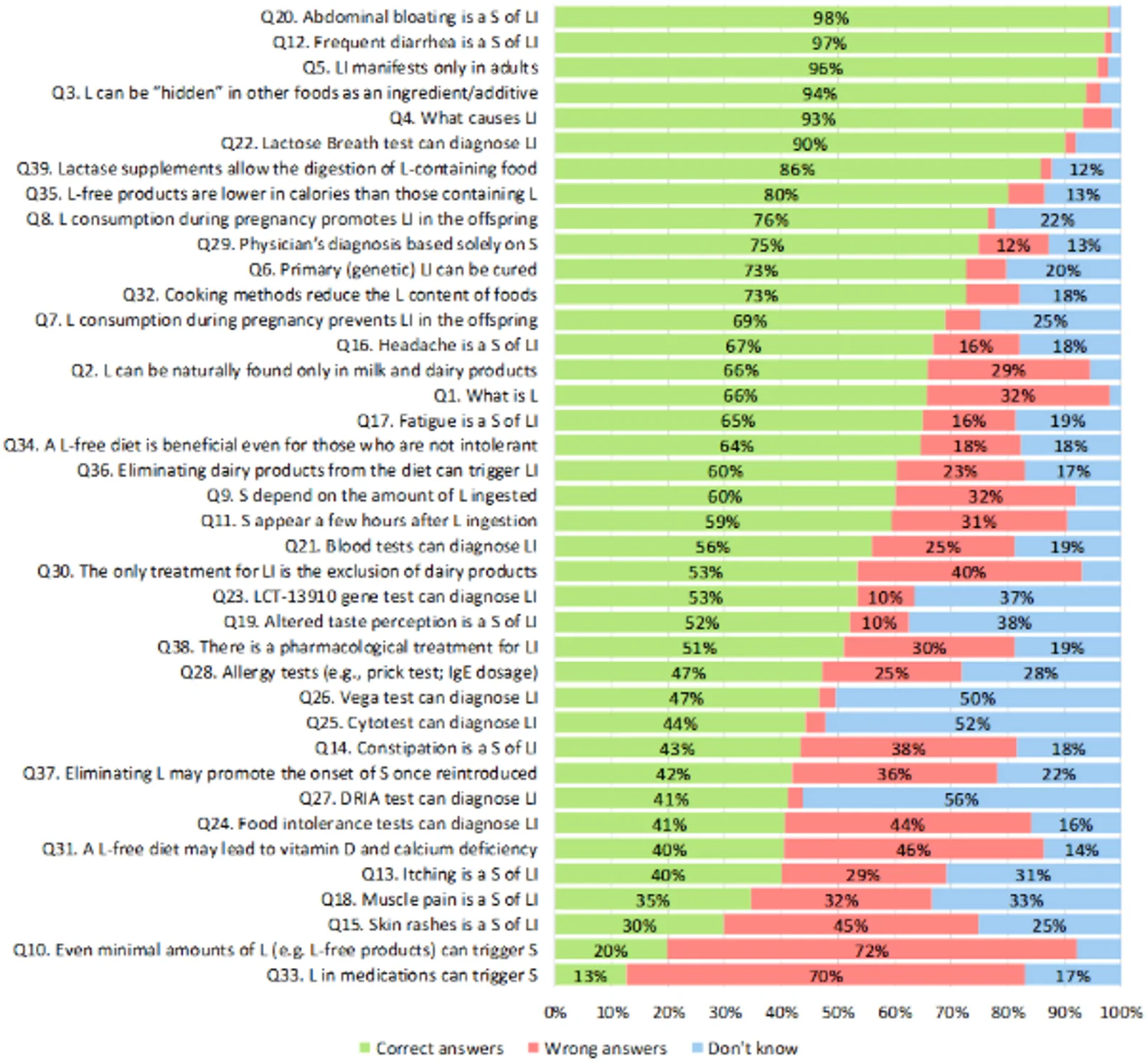
General knowledge about lactose intolerance among all respondents: responses ranked by percentage of correct answers to identify knowledge gaps

Figure 1 illustrates the overall percentage of correct, incorrect, and “I don’t know” responses across the total sample. The proportion of correct answers ranged from approximately 13% to 98%. Most respondents correctly identified that lactose can be “hidden” in foods as an ingredient/additive (94%), that LI is caused by lactase deficiency (93%), and that it can manifest at any age (96%). Furthermore, the majority recognized abdominal bloating (98%) and frequent diarrhea (97%) as typical symptoms, and the lactose breath test as a valid diagnostic tool (90%). Incorrect answers ranged from less than 1% to 72%. Notably, most respondents incorrectly believed that minimal amounts of lactose (e.g., in lactose-free products) can trigger symptoms (72%) and that lactose in medications can induce symptoms (70%). “I don’t know” responses ranged from 0% to 56%, with roughly half of the respondents uncertain about the validity of the Vega test (50%), Cytotest (52%), and DRIA test (56%).

Supplementary Figs. 2, 3 and 4 present the results stratified by LI status, nutrition expertise, and gender, respectively. Consistent with the overall findings, misconceptions were even more common among individuals with LI, with 82% incorrectly believing that minimal amounts of lactose in products and 78% that lactose in medications can trigger symptoms. Moreover, nearly half erroneously considered skin rashes a typical symptom (49%), dairy exclusion as the sole treatment for LI (49%), and temporary lactose elimination as a promoter of symptom onset upon reintroduction (45%) (Supplementary Fig. 2). Nutrition experts recorded the lowest proportion of “I don’t know” responses; however, important misconceptions remained. Specifically, 59% did not recognize that a lactose-free diet may lead to vitamin D and calcium deficiency, 49% failed to identify constipation as a typical symptom, and 74% incorrectly believed that trace amounts of lactose in medications can cause LI symptoms (Supplementary Fig. 3). Supplementary Table 1 provides the detailed distribution of responses (n, %) and highlights the correct answers for each of the 39 knowledge items.

**Fig. 2:**
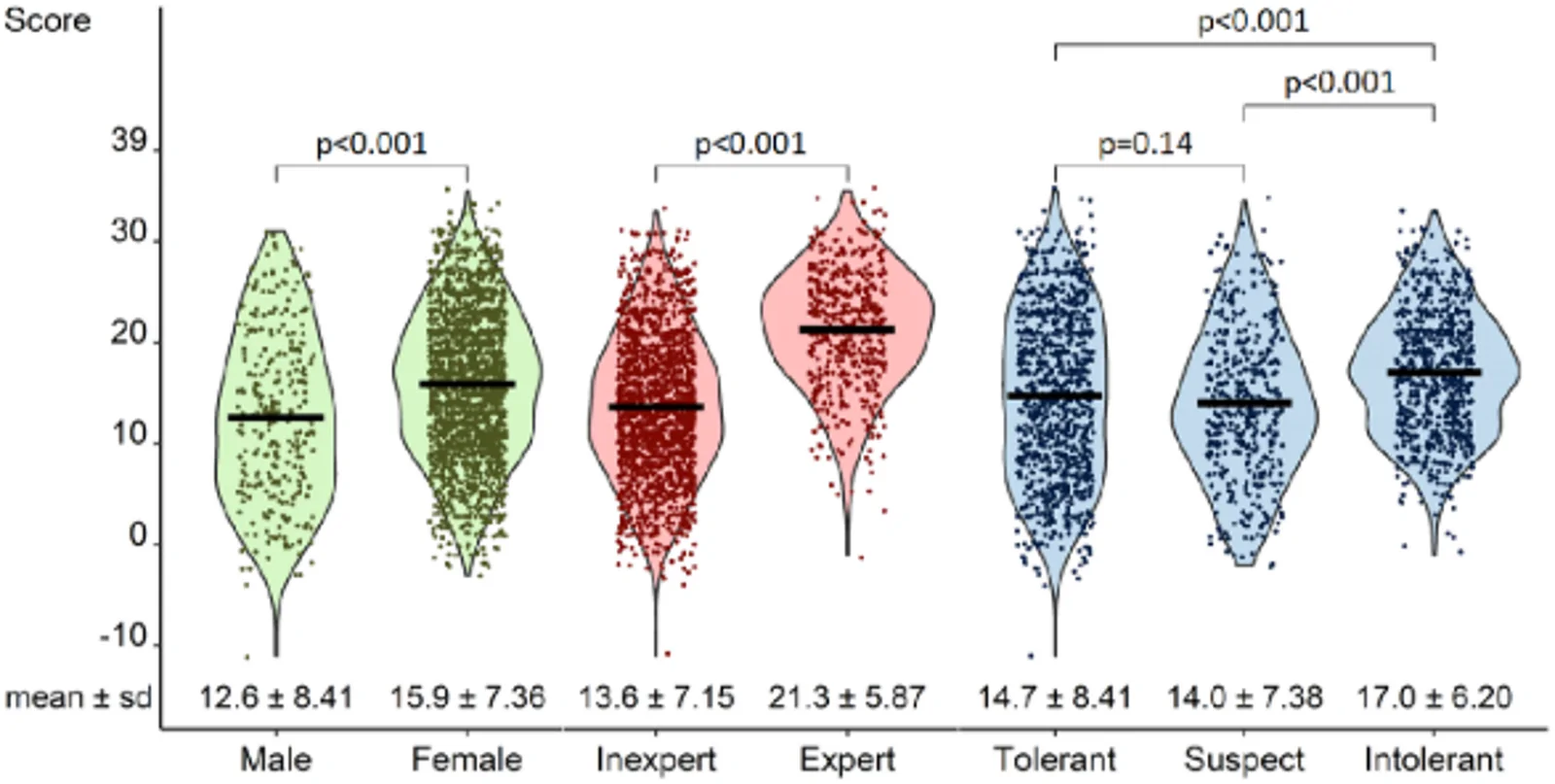
Overall knowledge about lactose intolerance by sex, nutritional expertise and lactose intolerance status

Table 2; Fig. 2 present the overall and module-specific knowledge scores for the total sample and stratified subgroups. In the total sample, the mean ± SD total knowledge score was 15.4 ± 7.6 (range: − 39 to + 39). Females (15.9 ± 7.4), lactose-intolerant individuals (17.0 ± 6.2), and nutrition experts (21.3 ± 5.9) scored significantly higher than their respective counterparts (*p* < 0.001) across overall scores and all four individual modules. However, the difference in Module 2 (“Symptoms”) between lactose-intolerant and lactose-tolerant respondents was not statistically significant. The “Definition” module attained the highest scores across all groups. In contrast, Module 2 (“Symptoms”, range: -12 to + 12) and Module 4 (“Management”, range: − 10 to + 10) recorded the lowest knowledge scores across all groups: total sample (3.4 ± 2.9 and 2.8 ± 2.4, respectively), females (3.5 ± 2.9 and 2.9 ± 2.4, respectively), lactose-intolerant individuals (3.5 ± 2.8 and 3.2 ± 2.2, respectively), and nutrition experts (4.9 ± 2.9 and 3.7 ± 2.2, respectively).

**Table 2:** Overall and module-specific knowledge scores for the total sample and stratified by gender, Lactose Intolerance status and nutritional expertise

	Total	Overall knowledge score#		Module 1 Definition score#		Module 2 Symptoms score#		Module 3 Diagnosis score#		Module 4 Management score#	
	N	Mean ± SD	*P*-value	Mean ± SD	*P*-value	Mean ± SD	*P*-value	Mean ± SD	*P*-value	Mean ± SD	*P*-value
*ALL*	2025	15.4 ± 7.6		5.5 ± 2.2		3.4 ± 2.9		3.7 ± 3.4		2.8 ± 2.4	
*Gender* ^*$*^											
Male	284	12.6 ± 8.4	Ref.	4.8 ± 2.5	Ref.	2.9 ± 3.0	Ref.	2.8 ± 3.5	Ref.	2.1 ± 2.6	Ref.
Female	1730	15.9 ± 7.4	< 0.0001	5.6 ± 2.1	< 0.0001	3.5 ± 2.9		3.8 ± 3.4	< 0.0001	2.9 ± 2.4	< 0.0001
*Lactose intolerance*											
No	913	14.7 ± 8.4	Ref.	5.3 ± 2.3	Ref.	3.3 ± 3.0	Ref.	3.4 ± 3.6	Ref.	2.6 ± 2.5	Ref.
Suspected	401	14.0 ± 7.4	0.14	5.4 ± 2.3	0.51	3.4 ± 3.0	0.74	2.6 ± 3.2	< 0.0001	2.6 ± 2.5	0.99
Confirmed	711	17.0 ± 6.2	< 0.0001	5.8 ± 2.0	**< **0.0001	3.5 ± 2.8	0.33	4.6 ± 3.0	**<** 0.0001	3.2 ± 2.2	< 0.0001
*Nutrition expert*											
No°	1558	13.6 ± 7.2	Ref.	5.1 ± 2.2	Ref.	3.0 ± 2.8	Ref.	3.0 ± 3.3	Ref.	2.6 ± 2.4	Ref.
Yes	467	21.3 ± 5.9	**< 0.0001**	6.6 ± 1.6	**< 0.0001**	4.9 ± 2.9	**< 0.0001**	6.1 ± 2.8	**< 0.0001**	3.7 ± 2.2	< 0.0001

Abbreviations. SD: Standard Deviation; Ref: Reference

^$^11 respondents preferred not to specify their gender

°Include 550 respondents interested in nutrition topics

#Total knowledge score: -39 to + 39; Module 1: -8 to + 8; Module 2: -12 to + 12; Module 3: -9 to + 9; Module 4: -10 to + 10

*P*-values based on Student’s t-test

Table 3 details respondents’ knowledge levels and their associations with demographic characteristics. Overall, 36.6% (741) of respondents were classified as having low knowledge, 32.2% (653) as having intermediate knowledge, and 31.2% (631) as having high knowledge about LI. The proportion of respondents with high knowledge was greater among females (32.7%; 566, OR 1.49, 95% CI: 1.15–1.93, *p* = 0.003). Age was not consistently associated with knowledge, though young adults (18–34 years) had the highest proportion of respondents with high knowledge (36.5%; 274). Geographically, knowledge was higher in the North-East of Italy (42.9%; 120), a finding confirmed in multivariable analysis (OR 1.64, 95% CI: 1.25–2.16, *p* = 0.0004). Higher education was strongly associated with knowledge, increasing progressively from high school or less (19.3% knowledgeable) to bachelor’s (36.4%), master’s (37.4%), and post-graduate/PhD degrees (38.5%). In multivariable analysis, holding a master’s degree (OR 1.36, 95% CI: 1.08–1.71, *p* = 0.009) or a post-graduate/PhD degree (OR 2.34, 95% CI: 1.69–3.25, *p* < 0.0001) remained significantly associated with higher knowledge. Finally, knowledge was significantly higher among lactose-intolerant individuals compared to tolerant and suspected ones (34.5% vs. 31.9% and 23.7%, respectively; OR 4.08, 95% CI: 3.24–5.14, *p* < 0.0001), while nutrition expertise showed the strongest overall association with high knowledge (OR 10.8, 95% CI: 8.30–14.2, *p* < 0.0001).

**Table 3:** Association between respondents’ characteristics and overall knowledge score about lactose intolerance

	Total	Low knowledge	Intermediate knowledge	High knowledge		Univariable analysis		Multivariable analysis	
	*N*	*N* (row %)	*N* (row %)	*N* (row %)	*P*-value†	OR (95% CI)	*P*-value^‡^	OR (95% CI)	*P*-value^‡^
*ALL*	2025	741 (36.6)	653 (32.2)	631 (31.2)					
*Gender* ^*$*^									
Male	284	142 (50.0)	78 (27.5)	64 (22.5)		1.00		1.00	
Female	1730	595 (34.4)	569 (32.9)	566 (32.7)	< 0.0001	1.83 (1.44–2.32)	< 0.0001	1.49 (1.15–1.93)	0.003
*Age*									
18–34	750	247 (32.9)	229 (30.5)	274 (36.5)		1.00		1.00	
35–64	1185	457 (38.6)	388 (32.7)	340 (28.7)		0.74 (0.62–0.88)	0.0004	0.81 (0.64–1.01)	0.066
65+	90	37 (41.1)	36 (40.0)	17 (18.9)	0.0004	0.58 (0.38–0.86)	0.008	0.53 (0.28–1.01)	0.054
*Italian region*									
North-West	801	385 (48.1)	221 (27.6)	195 (24.3)		1.00		1.00	
North-East	280	64 (22.9)	96 (34.3)	120 (42.9)		2.74 (2.12–3.53)	< 0.0001	1.64 (1.25–2.16)	0.0004
Central	481	124 (25.8)	175 (36.4)	182 (37.8)		2.28 (1.85–2.82)	< 0.0001	1.26 (1.00-1.59)	0.052
South	320	113 (35.3)	107 (33.4)	100 (31.3)		1.60 (1.26–2.03)	0.0001	0.98 (0.75–1.27)	0.88
Islands	143	55 (38.5)	54 (37.8)	34 (23.8)	< 0.0001	1.27 (0.91–1.77)	0.16	1.06 (0.75–1.50)	0.76
*Education level*									
High school or less	685	319 (46.6)	234 (34.2)	132 (19.3)		1.00		1.00	
Bachelor’s degree	450	151 (33.6)	135 (30.0)	164 (36.4)		1.97 (1.58–2.46)	< 0.0001	1.23 (0.96–1.58)	0.11
Master’s degree	698	214 (30.7)	223 (31.9)	261 (37.4)		2.15 (1.76–2.61)	< 0.0001	1.36 (1.08–1.71)	0.009
Post-graduate /PhD	192	57 (29.7)	61 (31.8)	74 (38.5)	< 0.0001	2.25 (1.67–3.03)	< 0.0001	2.34 (1.69–3.25)	< 0.0001
*Employment status*									
Student	216	90 (41.7)	67 (31.0)	59 (27.3)		1.00		1.00	
Employed	1541	555 (36.0)	479 (31.1)	507 (32.9)		1.30 (0.99–1.68)	0.06	0.96 (0.70–1.33)	0.81
Housewife	96	32 (33.3)	39 (40.6)	25 (26.0)		1.19 (0.76–1.85)	0.45	1.22 (0.73–2.03)	0.46
Retiree	92	39 (42.4)	38 (41.3)	15 (16.3)		0.80 (0.51–1.26)	0.34	1.09 (0.55–2.17)	0.80
Unemployed	80	25 (31.3)	30 (37.5)	25 (31.3)	0.01	1.38 (0.86–2.22)	0.18	0.99 (0.59–1.66)	0.97
*Marital status*									
Married	932	341 (36.6)	312 (33.5)	279 (29.9)		1.00		1.00	
Widow(er)	22	7 (31.8)	10 (45.5)	5 (22.7)		0.96 (0.44–2.10)	0.93	1.23 (0.52–2.91)	0.63
Divorced	121	57 (47.1)	32 (26.4)	32 (26.4)		0.72 (0.50–1.02)	0.06	0.78 (0.54–1.14)	0.20
Single	950	336 (35.4)	299 (31.5)	315 (33.2)	0.11	1.10 (0.93–1.30)	0.24	0.93 (0.75–1.15)	0.49
*Lactose intolerance*									
No	913	378 (41.4)	244 (26.7)	291 (31.9)		1.00		1.00	
Suspected	401	174 (43.4)	132 (32.9)	95 (23.7)		0.81 (0.65-1.00)	0.050	1.60 (1.25–2.05)	0.0002
Confirmed	711	189 (26.6)	277 (39.0)	245 (34.5)	< 0.0001	1.48 (1.24–1.77)	< 0.0001	4.08 (3.24–5.14)	< 0.0001
*Nutrition expert*									
No°	1558	704 (45.2)	518 (33.2)	336 (21.6)		1.00		1.00	
Yes	467	37 ( 7.9)	135 (28.9)	295 (63.2)	< 0.0001	6.82 (5.50–8.45)	< 0.0001	10.8 (8.30–14.2)	< 0.0001

^$^11 responders preferred not to specify their gender

°Include 550 respondents interested in nutrition topics

Odds Ratios (ORs) and 95% confidence intervals (CI) obtained from univariable and multivariable ordinal logistic regression with all variables fitted simultaneously

†* P*-value based on Chi-square test ‡ P-value based on Wald test from ordinal logistic regression

Abbreviations. LI: Lactose intolerance; L: Lactose; S: Symptom(s)

Percentages under 10% are not reported in the graphic

Total knowledge score can range from − 39 to + 39

*P*-values based on Student’s t-test

Statistically significant values are expressed in bold

## Discussion

The present survey provides valuable insights into the knowledge of LI and its self-reported prevalence among Italian adults. Analysis of responses identified both areas of well-established knowledge and persistent knowledge gaps. Overall, 31.2% of respondents were classified as having high knowledge, 32.2% demonstrated intermediate knowledge, and 36.6% exhibited low knowledge.

The “Definition” module attained the highest performance across all respondent groups, indicating a solid understanding of the basic LI principles. Notably, 94% of participants correctly identified the presence of “hidden” lactose in non-dairy processed foods (Q3), confirming widespread awareness of its use as a functional industrial additive [2, 22]. As expected, nutrition experts and individuals with confirmed LI achieved the highest overall knowledge scores, supporting the role of professional training and personal experience as primary drivers of health literacy. Among individuals with LI, higher knowledge levels also coincided with a higher educational background, highlighting the potential contribution of general literacy to LI self-management. Multivariable analysis revealed no significant association between age and LI knowledge, although the slightly higher scores among younger respondents may suggest a generational difference potentially related to digital health information access [35, 36].

Despite a solid understanding of basic definitions, knowledge concerning LI management was notably weaker across all groups, including nutrition experts. The most alarming knowledge deficit pertained to dietary restrictions and associated nutritional risks: 59% of nutrition experts incorrectly believed that a lactose-free diet carries no risk of calcium and vitamin D deficiencies (Q31). This widespread misconception may be reinforced by the limited emphasis placed in current clinical guidelines on ensuring adequate intake of these nutrients in individuals with LI. Consistently, Dominici et al. (2025) reported that healthcare workers frequently recommend total exclusion of dairy products [13], which may increase the risk of inadequate calcium and vitamin D intake and adversely affect bone health [12]. This finding highlights the need to distinguish lactose restriction from dairy elimination, as nutritional adequacy can generally be maintained through an appropriately balanced diet including tolerated dairy products, non-dairy or lactose-free alternatives.

Uncertainty regarding physiological tolerance thresholds emerged across multiple survey domains, including pharmaceutical excipients, trace residual lactose in foods, lactose reintroduction and microbial adaptation. Regarding lactose-containing medications (Q33), lactose is commonly utilized as a pharmaceutical excipient, but the amounts involved are typically negligible (milligram range) and generally insufficient to induce symptoms in most individuals with LI [2, 5, 6]. Nevertheless, responses suggested a perception that lactose-containing medications can trigger symptoms. As LI symptoms are dose-dependent, cumulative lactose exposure from multiple sources may exceed the individual tolerance threshold in susceptible subjects. Furthermore, symptoms occurring during pharmacological treatment may instead reflect drug-related adverse effects, coexisting gastrointestinal disorders, gut microbiota alterations, or patients’ attribution rather than the lactose content itself [5].

Similarly, 72% of respondents incorrectly identified commercial lactose-free products as potential symptom triggers (Q10). Most individuals with LI can tolerate small amounts of lactose, approximately 3–5 g in a single-dose, due to residual lactase activity. Consequently, the trace amounts remaining in commercial products labeled “lactose-free”, typically 0.1% or 0.01% (1000 mg/kg or 100 mg/kg, respectively), are generally considered physiologically safe [5]. Nevertheless, the high proportion of incorrect responses suggests a widespread belief that even trace amounts of lactose can trigger symptoms [4, 37–39]. Symptoms attributed to these products may instead reflect individual variability, coexisting gastrointestinal disorders, altered gut microbiota, or heightened symptom perception.

Approximately 45% of respondents also answered incorrectly regarding symptom onset upon lactose reintroduction (Q37), indicating limited awareness of mechanisms that may modulate lactose tolerance independently of lactase activity. While lactase is not an inducible enzyme, gradual lactose reintroduction may promote colonic microbiota adaptation, enhancing short-chain fatty acid fermentation and increasing individual tolerance [40]. However, given the substantial individual variability, these mechanisms should not be interpreted as guaranteeing clinical benefit.

The survey also reveals marked differences in knowledge of symptomatology. Recognition of typical gastrointestinal symptoms associated with lactose malabsorption was high, particularly for abdominal bloating (Q20: 98%) and frequent diarrhea (Q12: 97%), whereas awareness dropped sharply for less typical manifestations such as constipation (Q14: 43%) [41]. Extraintestinal manifestations exhibited even lower recognition, with only 30% to 40% of respondents correctly classifying systemic symptoms such as itching (Q13), muscle pain (Q18), or skin rashes (Q15). Although such symptoms are frequently reported by patients, robust pathophysiological mechanisms directly linking lactose ingestion to extraintestinal symptoms remain unproven in scientific literature [4, 22, 42, 43]. Accordingly, these findings should be interpreted in light of the current evidence rather than as excluding the possibility that individual patients may report such symptoms. More broadly, some items in the symptoms and management modules address clinically nuanced topics in which evidence remains evolving, requiring distinction between well-established evidence, plausible biological mechanisms, and patient-reported perceptions.

The highest rates of “don’t know” responses were recorded for questions concerning unvalidated diagnostic methods. While spontaneous recognition of unvalidated diagnostic procedures (e.g., Vega test, Cytotest, DRIA test) was low, 44% of participants explicitly endorsed general food intolerance tests as valid diagnostic tools (Q24). This misconception may be reinforced by the commercial availability and promotion of non-evidence-based testing and by limited standardized patient guidance, posing a significant risk of misdiagnosis and inappropriate dietary restriction [44].

A total of 35.1% of participants self-reported a clinically confirmed diagnosis of LI based on validated diagnostic testing (lactose breath test, LCT-13910 genetic test, or both). This finding aligns closely with epidemiological data for Italy, where lactose malabsorption affects approximately 50% of the general population, with 30% to 40% developing overt clinical symptoms. Our findings also reflected the established North-South geographical gradient in lactase non-persistence, with higher self-reported LI rates in Southern Italy and the Islands [29]. This regional distribution is consistent with ancestral Mediterranean genetic variants and historical selection pressures regarding lactase persistence [18]. However, the higher rates of LI in the Southern regions were not consistent with greater knowledge scores. In fact, respondents from North-Eastern regions showed the highest knowledge levels, pointing out that LI prevalence does not necessarily translate into greater knowledge of the condition.

Overall, these findings have important implications for the clinical and dietary management of LI in Italy. By assessing knowledge among the general population, individuals with LI, and nutrition experts, this survey identified critical information gaps across key target groups. These insights provide the foundation for targeted educational materials for both the public and health professionals. To address these gaps, the established inter-society working group will draft a consensus document grounded in scientific evidence to guide proper diagnosis, the safe use of lactose-free products and pharmaceutical excipients, and personalized dietary management, while discouraging unnecessary dietary restrictions.

This study has several strengths, alongside some limitations. The online distribution of the survey allowed us to reach a large sample across Italy, supported by the country’s high internet penetration [35]. This approach enabled us to successfully exceed our planned sample size, permitted data stratification and provided valuable insights into specific subgroups. Furthermore, although the survey was non-validated, the extensive number of items included allowed for a comprehensive assessment of different domains of LI knowledge.

On the other hand, certain limitations must be acknowledged. First, all data, including LI diagnosis and diagnostic testing, were self-reported, introducing the possibility of misclassification as well as recall and reporting biases. Second, voluntary online recruitment may have introduced selection bias by attracting individuals who were more health-conscious or personally interested in LI. Third, the sample exhibits a significant demographic and professional imbalance, with an overrepresentation of women, highly educated participants and nutrition experts [45]. This distribution likely reflects the recruitment strategy and may limit the generalizability of the findings to the broader Italian population, as these groups typically have greater health awareness. Finally, some potentially relevant confounders, such as income, were not assessed, and the questionnaire’s length may have affected completion rates.

## Conclusion

In conclusion, LI knowledge is associated with multiple factors, including gender, geographical residence, education level and background, and LI status. Our findings highlight the need for targeted educational interventions and ad-hoc documentation to improve knowledge of LI, thereby preventing misconceptions that can negatively impact both short and long-term management of the condition. Addressing the nutritional gaps linked to LI requires targeted dietary guidelines and dynamic databases providing accurate data on the lactose content of generic foods and recipes. Our findings on LI prevalence serve as a stimulus for the food industry to expand and certify lactose-free products, thereby enhancing nutritional security and dietary diversity for this population.

## Supplementary Information

Below is the link to the electronic supplementary material.

Supplementary Material 1

## Data Availability

The datasets generated during and/or analyzed during the current study are available from the corresponding author on reasonable request.
