# Supplementary material for: Knowledge of lactose intolerance in Italian adults: a cross‑sectional assessment
Source: Eur J Nutr. 2026 Sep 24;65(7):258. doi: 10.1007/s00394-026-04106-4 (PMC13612621; doi:10.1007/s00394-026-04106-4)
Supplement: Supplementary file 1 [file 394_2026_4106_MOESM1_ESM.pdf]

Supplementary Figure 1. Flow-chart of survey design process

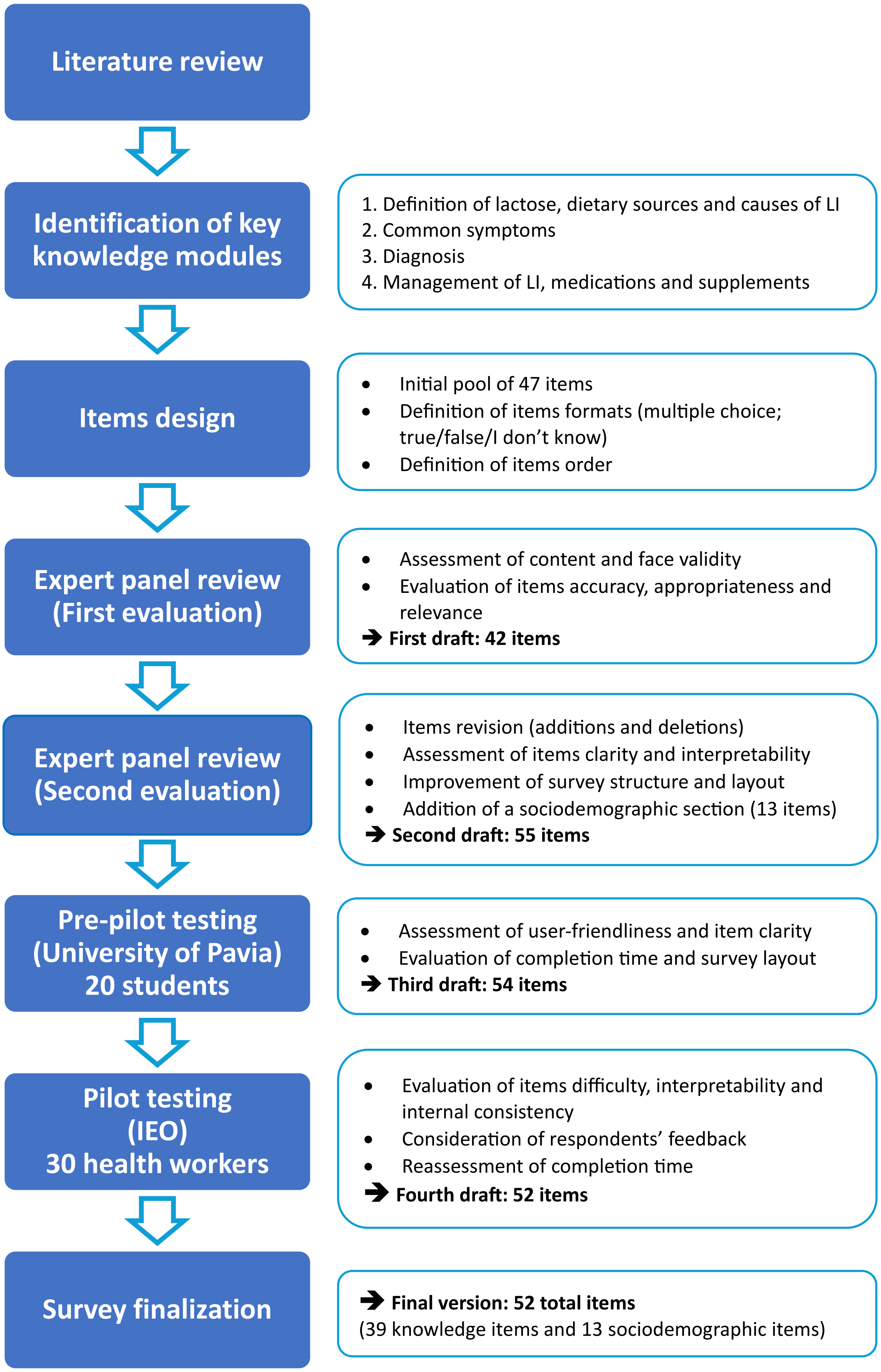

Abbreviations. LI: Lactose intolerance; IEO: European Institute of Oncology

Supplementary Figure 2. General knowledge about lactose intolerance: differences in responses according to lactose intolerance status

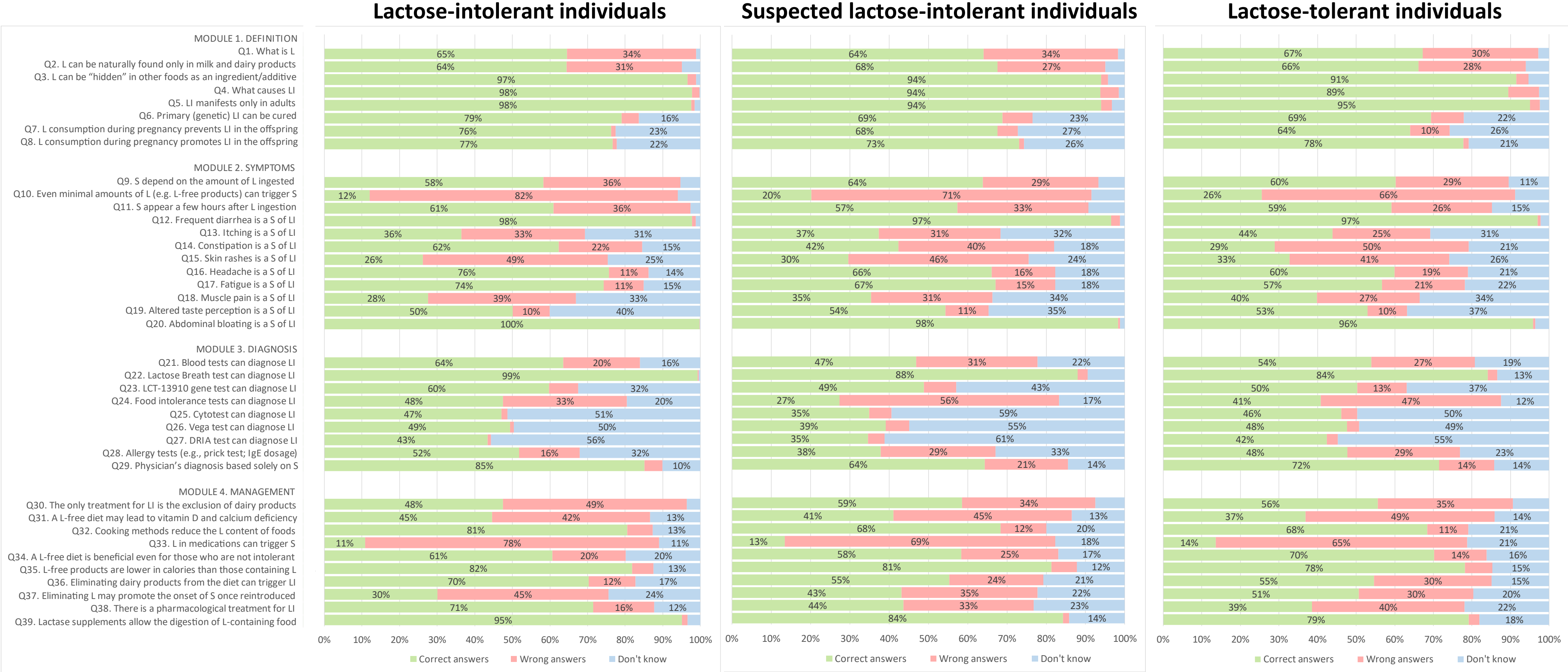

Abbreviations. LI: Lactose intolerance; L: Lactose; S: Symptom(s).

Percentages under 10% are not reported in the graphic

Supplementary Figure 3. General knowledge about lactose intolerance: differences in responses according to nutrition-related education

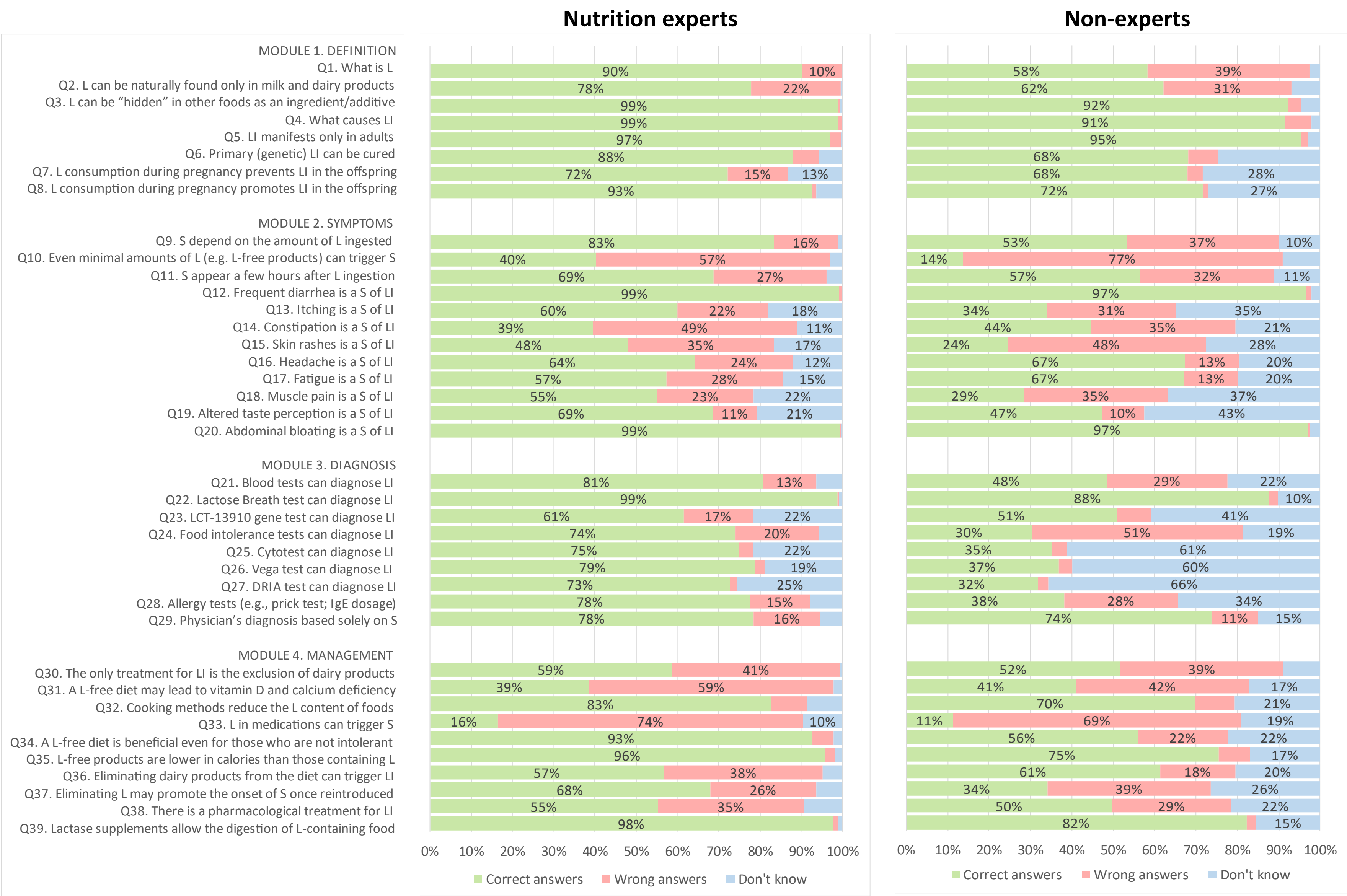

Abbreviations. LI: Lactose intolerance; L: Lactose; S: Symptom(s).

Percentages under 10% are not reported in the graphic

Supplementary Figure 4. General knowledge about lactose intolerance: differences in responses by gender

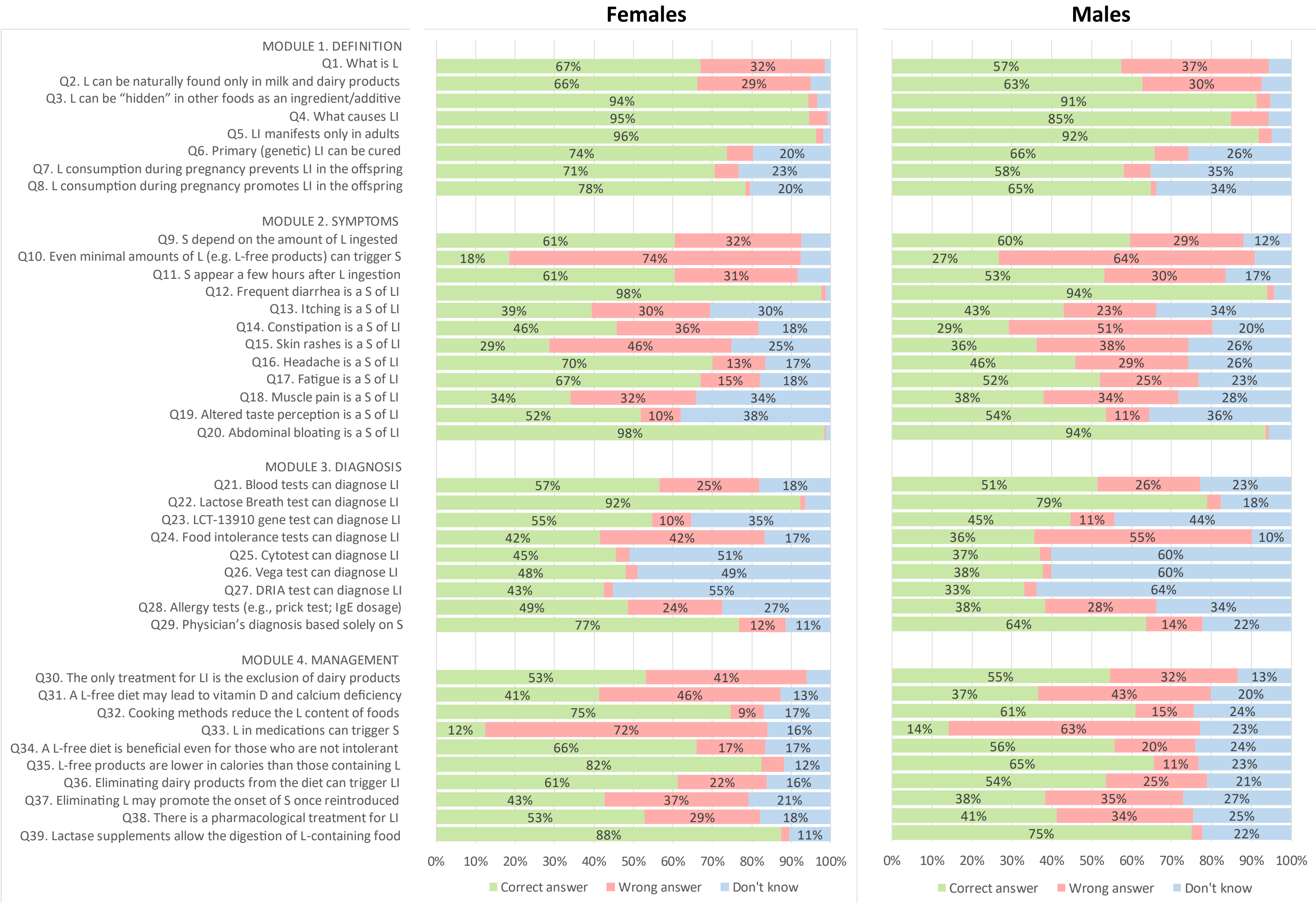

Abbreviations. LI: Lactose intolerance; L: Lactose; S: Symptom(s).

Percentages under 10% are not reported in the graphic

Supplementary Table 1. Detailed distribution of responses (n, %) for each of the 39 knowledge items.

All	Total N=2025 N (col %)	Lactose Tolerant N=913 N (col %)	Suspect Intolerant N=401 N (col %)	Lactose Intolerant N=711 N (col %)	P-value	Inexpert N=1558 N (col %)	Nutrition Expert N=467 N (col %)	P-value	Male N=284 N (col %)	Female N=1730 N (col %)	P-value
**MODULE 1 – DEFINITION**											
**What is L?**											
Sugar found only in dairy products	1330 (65.7)	614 (67.3)	257 (64.1)	459 (64.6)		909 (58.3)	421 (90.1)		163 (57.4)	1160 (67.1)	
Sugar found only in cereals	1 (0.0)	0 (0.0)	0 (0.0)	1 (0.1)		1 (0.1)	0 (0.0)		0 (0.0)	1 (0.1)	
Sugar condensed in milk proteins	444 (21.9)	183 (20.0)	98 (24.4)	163 (22.9)		400 (25.7)	44 (9.4)		77 (27.1)	364 (21.0)	
Protein found in foods	210 (10.4)	91 (10.0)	39 (9.7)	80 (11.3)		208 (13.4)	2 (0.4)		28 (9.9)	181 (10.5)	
I don't know	40 (2.0)	25 (2.7)	7 (1.7)	8 (1.1)	0.17	40 (2.6)	0 (0.0)	**<.0001**	16 (5.6)	24 (1.4)	**<.0001**
**L can be naturally found only in milk and dairy products**											
True	1333 (65.8)	604 (66.2)	271 (67.6)	458 (64.4)		969 (62.2)	364 (77.9)		178 (62.7)	1146 (66.2)	
False	582 (28.7)	254 (27.8)	110 (27.4)	218 (30.7)		481 (30.9)	101 (21.6)		85 (29.9)	495 (28.6)	
I don't know	110 (5.4)	55 (6.0)	20 (5.0)	35 (4.9)	0.57	108 (6.9)	2 (0.4)	**<.0001**	21 (7.4)	89 (5.1)	0.24
**L can be "hidden" in other foods (besides milk and dairy) as an ingredient/additive**											
True	1899 (93.8)	835 (91.5)	377 (94.0)	687 (96.6)		1437 (92.2)	462 (98.9)		259 (91.2)	1631 (94.3)	
False	52 (2.6)	29 (3.2)	7 (1.7)	16 (2.3)		50 (3.2)	2 (0.4)		10 (3.5)	40 (2.3)	
I don't know	74 (3.7)	49 (5.4)	17 (4.2)	8 (1.1)	**0.0001**	71 (4.6)	3 (0.6)	**<.0001**	15 (5.3)	59 (3.4)	0.13
**LI is caused by**											
Allergy to lactose	41 (2.0)	26 (2.8)	9 (2.2)	6 (0.8)		40 (2.6)	1 (0.2)		11 (3.9)	30 (1.7)	
Allergy to milk proteins	46 (2.3)	36 (3.9)	5 (1.2)	5 (0.7)		44 (2.8)	2 (0.4)		9 (3.2)	36 (2.1)	
Reduced lactose digestion due to enzyme deficiency	1887 (93.2)	816 (89.4)	376 (93.8)	695 (97.7)		1425 (91.5)	462 (98.9)		241 (84.9)	1636 (94.6)	
Excessive intake of L	15 (0.7)	9 (1.0)	3 (0.7)	3 (0.4)		15 (1.0)	0 (0.0)		5 (1.8)	10 (0.6)	
Exclusion of L from the diet	4 (0.2)	2 (0.2)	2 (0.5)	0 (0.0)		2 (0.1)	2 (0.4)		2 (0.7)	2 (0.1)	
I don't know	32 (1.6)	24 (2.6)	6 (1.5)	2 (0.3)	**<.0001**	32 (2.1)	0 (0.0)	**<.0001**	16 (5.6)	16 (0.9)	**<.0001**
**LI manifests only in adults**											
True	40 (2.0)	23 (2.5)	11 (2.7)	6 (0.8)		26 (1.7)	14 (3.0)		9 (3.2)	31 (1.8)	
False	1939 (95.8)	868 (95.1)	377 (94.0)	694 (97.6)		1487 (95.4)	452 (96.8)		261 (91.9)	1667 (96.4)	
I don't know	46 (2.3)	22 (2.4)	13 (3.2)	11 (1.5)	**0.03**	45 (2.9)	1 (0.2)	**0.001**	14 (4.9)	32 (1.8)	**0.002**
**Primary (genetic) LI can be cured**											
True	141 (7.0)	77 (8.4)	31 (7.7)	33 (4.6)		112 (7.2)	29 (6.2)		24 (8.5)	116 (6.7)	
False	1472 (72.7)	634 (69.4)	276 (68.8)	562 (79.0)		1061 (68.1)	411 (88.0)		187 (65.8)	1276 (73.8)	
I don't know	412 (20.3)	202 (22.1)	94 (23.4)	116 (16.3)	**0.0001**	385 (24.7)	27 (5.8)	**<.0001**	73 (25.7)	338 (19.5)	**0.02**
**L consumption during pregnancy prevents LI in the offspring**											
True	124 (6.1)	94 (10.3)	21 (5.2)	9 (1.3)		56 (3.6)	68 (14.6)		19 (6.7)	104 (6.0)	
False	1397 (69.0)	584 (64.0)	271 (67.6)	542 (76.2)		1060 (68.0)	337 (72.2)		165 (58.1)	1223 (70.7)	
I don't know	504 (24.9)	235 (25.7)	109 (27.2)	160 (22.5)	**<.0001**	442 (28.4)	62 (13.3)	**<.0001**	100 (35.2)	403 (23.3)	**<.0001**
**L consumption during pregnancy promotes LI in the offspring**											
True	26 (1.3)	13 (1.4)	5 (1.2)	8 (1.1)		22 (1.4)	4 (0.9)		4 (1.4)	22 (1.3)	
False	1548 (76.4)	710 (77.8)	293 (73.1)	545 (76.7)		1115 (71.6)	433 (92.7)		184 (64.8)	1356 (78.4)	
I don't know	451 (22.3)	190 (20.8)	103 (25.7)	158 (22.2)	0.40	421 (27.0)	30 (6.4)	**<.0001**	96 (33.8)	352 (20.3)	**<.0001**
**MODULE 2 – SYMPTOMS**											
**Symptoms depend on the amount of L ingested with the diet**											
True	1220 (60.2)	550 (60.2)	256 (63.8)	414 (58.2)		831 (53.3)	389 (83.3)		169 (59.5)	1047 (60.5)	
False	644 (31.8)	267 (29.2)	118 (29.4)	259 (36.4)		571 (36.6)	73 (15.6)		81 (28.5)	557 (32.2)	
I don't know	161 (8.0)	96 (10.5)	27 (6.7)	38 (5.3)	**0.0001**	156 (10.0)	5 (1.1)	**<.0001**	34 (12.0)	126 (7.3)	**0.02**
**Even minimal amounts of L (e.g., L-free products) can trigger symptoms**											
True	1468 (72.5)	600 (65.7)	286 (71.3)	582 (81.9)		1204 (77.3)	264 (56.5)		182 (64.1)	1279 (73.9)	
False	400 (19.8)	233 (25.5)	81 (20.2)	86 (12.1)		212 (13.6)	188 (40.3)		76 (26.8)	320 (18.5)	
I don't know	157 (7.8)	80 (8.8)	34 (8.5)	43 (6.0)	**<.0001**	142 (9.1)	15 (3.2)	**<.0001**	26 (9.2)	131 (7.6)	**0.002**
**Symptoms appear a few hours after L ingestion**											
True	1203 (59.4)	540 (59.1)	230 (57.4)	433 (60.9)		882 (56.6)	321 (68.7)		151 (53.2)	1047 (60.5)	
False	631 (31.2)	238 (26.1)	134 (33.4)	259 (36.4)		503 (32.3)	128 (27.4)		86 (30.3)	539 (31.2)	
I don't know	191 (9.4)	135 (14.8)	37 (9.2)	19 (2.7)	**<.0001**	173 (11.1)	18 (3.9)	**<.0001**	47 (16.5)	144 (8.3)	**<.0001**
**Which symptoms are associated with LI?**											
Frequent diarrhea											
True	1968 (97.2)	886 (97.0)	387 (96.5)	695 (97.7)		1505 (96.6)	463 (99.1)		267 (94.0)	1690 (97.7)	
False	23 (1.1)	7 (0.8)	9 (2.2)	7 (1.0)		19 (1.2)	4 (0.9)		5 (1.8)	18 (1.0)	
I don't know	34 (1.7)	20 (2.2)	5 (1.2)	9 (1.3)	0.08	34 (2.2)	0 (0.0)	**0.004**	12 (4.2)	22 (1.3)	**0.001**
Itching											
True	589 (29.1)	231 (25.3)	124 (30.9)	234 (32.9)		487 (31.3)	102 (21.8)		66 (23.2)	520 (30.1)	
False	810 (40.0)	401 (43.9)	150 (37.4)	259 (36.4)		530 (34.0)	280 (60.0)		122 (43.0)	683 (39.5)	
I don't know	626 (30.9)	281 (30.8)	127 (31.7)	218 (30.7)	**0.005**	541 (34.7)	85 (18.2)	**<.0001**	96 (33.8)	527 (30.5)	0.06
Constipation											
True	877 (43.3)	264 (28.9)	170 (42.4)	443 (62.3)		693 (44.5)	184 (39.4)		83 (29.2)	790 (45.7)	
False	776 (38.3)	459 (50.3)	159 (39.7)	158 (22.2)		545 (35.0)	231 (49.5)		145 (51.1)	625 (36.1)	
I don't know	372 (18.4)	190 (20.8)	72 (18.0)	110 (15.5)	**<.0001**	320 (20.5)	52 (11.1)	**<.0001**	56 (19.7)	315 (18.2)	**<.0001**
Skin rashes											
True	912 (45.0)	378 (41.4)	184 (45.9)	350 (49.2)		747 (47.9)	165 (35.3)		108 (38.0)	799 (46.2)	
False	604 (29.8)	299 (32.7)	119 (29.7)	186 (26.2)		380 (24.4)	224 (48.0)		103 (36.3)	496 (28.7)	
I don't know	509 (25.1)	236 (25.8)	98 (24.4)	175 (24.6)	**0.02**	431 (27.7)	78 (16.7)	**<.0001**	73 (25.7)	435 (25.1)	**0.02**
Headache											
True	1350 (66.7)	547 (59.9)	265 (66.1)	538 (75.7)		1050 (67.4)	300 (64.2)		130 (45.8)	1212 (70.1)	
False	314 (15.5)	174 (19.1)	65 (16.2)	75 (10.5)		203 (13.0)	111 (23.8)		81 (28.5)	232 (13.4)	
I don't know	361 (17.8)	192 (21.0)	71 (17.7)	98 (13.8)	**<.0001**	305 (19.6)	56 (12.0)	**<.0001**	73 (25.7)	286 (16.5)	**<.0001**
Fatigue											
True	1315 (64.9)	518 (56.7)	269 (67.1)	528 (74.3)		1047 (67.2)	268 (57.4)		148 (52.1)	1160 (67.1)	
False	333 (16.4)	196 (21.5)	61 (15.2)	76 (10.7)		202 (13.0)	131 (28.1)		70 (24.6)	260 (15.0)	
I don't know	377 (18.6)	199 (21.8)	71 (17.7)	107 (15.0)	**<.0001**	309 (19.8)	68 (14.6)	**<.0001**	66 (23.2)	310 (17.9)	**<.0001**
Muscle pain											
True	647 (32.0)	243 (26.6)	124 (30.9)	280 (39.4)		538 (34.5)	109 (23.3)		96 (33.8)	549 (31.7)	
False	702 (34.7)	364 (39.9)	142 (35.4)	196 (27.6)		445 (28.6)	257 (55.0)		108 (38.0)	589 (34.0)	
I don't know	676 (33.4)	306 (33.5)	135 (33.7)	235 (33.1)	**<.0001**	575 (36.9)	101 (21.6)	**<.0001**	80 (28.2)	592 (34.2)	0.13
Altered taste perception											
True	208 (10.3)	94 (10.3)	44 (11.0)	70 (9.8)		158 (10.1)	50 (10.7)		31 (10.9)	177 (10.2)	
False	1057 (52.2)	483 (52.9)	218 (54.4)	356 (50.1)		737 (47.3)	320 (68.5)		152 (53.5)	897 (51.8)	
I don't know	760 (37.5)	336 (36.8)	139 (34.7)	285 (40.1)	0.46	663 (42.6)	97 (20.8)	**<.0001**	101 (35.6)	656 (37.9)	0.74
Abdominal bloating											
True	1978 (97.7)	875 (95.8)	394 (98.3)	709 (99.7)		1514 (97.2)	464 (99.4)		266 (93.7)	1701 (98.3)	
False	8 (0.4)	5 (0.5)	2 (0.5)	1 (0.1)		6 (0.4)	2 (0.4)		2 (0.7)	6 (0.3)	
I don't know	39 (1.9)	33 (3.6)	5 (1.2)	1 (0.1)	**<.0001**	38 (2.4)	1 (0.2)	**0.01**	16 (5.6)	23 (1.3)	**<.0001**
**MODULE 3 – DIAGNOSIS**											
**Which tests can be used to diagnose LI?**											
Blood test											
True	514 (25.4)	245 (26.8)	124 (30.9)	145 (20.4)		454 (29.1)	60 (12.8)		73 (25.7)	438 (25.3)	
False	1132 (55.9)	492 (53.9)	188 (46.9)	452 (63.6)		755 (48.5)	377 (80.7)		146 (51.4)	979 (56.6)	
I don't know	379 (18.7)	176 (19.3)	89 (22.2)	114 (16.0)	**<.0001**	349 (22.4)	30 (6.4)	**<.0001**	65 (22.9)	313 (18.1)	0.12

Lactose breath test											
True	1827 (90.2)	768 (84.1)	353 (88.0)	706 (99.3)		1366 (87.7)	461 (98.7)		224 (78.9)	1595 (92.2)	
False	34 (1.7)	22 (2.4)	10 (2.5)	2 (0.3)		32 (2.1)	2 (0.4)		10 (3.5)	23 (1.3)	
I don't know	164 (8.1)	123 (13.5)	38 (9.5)	3 (0.4)	**<.0001**	160 (10.3)	4 (0.9)	**<.0001**	50 (17.6)	112 (6.5)	**<.0001**
LCT-13910 gene test											
True	1080 (53.3)	459 (50.3)	196 (48.9)	425 (59.8)		793 (50.9)	287 (61.5)		127 (44.7)	947 (54.7)	
False	205 (10.1)	117 (12.8)	33 (8.2)	55 (7.7)		127 (8.2)	78 (16.7)		31 (10.9)	173 (10.0)	
I don't know	740 (36.5)	337 (36.9)	172 (42.9)	231 (32.5)	**<.0001**	638 (40.9)	102 (21.8)	**<.0001**	126 (44.4)	610 (35.3)	**0.006**
Food intolerance tests											
True	884 (43.7)	426 (46.7)	224 (55.9)	234 (32.9)		790 (50.7)	94 (20.1)		155 (54.6)	721 (41.7)	
False	821 (40.5)	373 (40.9)	110 (27.4)	338 (47.5)		475 (30.5)	346 (74.1)		101 (35.6)	718 (41.5)	
I don't know	320 (15.8)	114 (12.5)	67 (16.7)	139 (19.5)	**<.0001**	293 (18.8)	27 (5.8)	**<.0001**	28 (9.9)	291 (16.8)	**<.0001**
Cytotest											
True	72 (3.6)	38 (4.2)	23 (5.7)	11 (1.5)		56 (3.6)	16 (3.4)		8 (2.8)	62 (3.6)	
False	896 (44.2)	421 (46.1)	140 (34.9)	335 (47.1)		547 (35.1)	349 (74.7)		105 (37.0)	787 (45.5)	
I don't know	1057 (52.2)	454 (49.7)	238 (59.4)	365 (51.3)	**<.0001**	955 (61.3)	102 (21.8)	**<.0001**	171 (60.2)	881 (50.9)	**0.01**
Vega test											
True	60 (3.0)	29 (3.2)	24 (6.0)	7 (1.0)		49 (3.1)	11 (2.4)		6 (2.1)	53 (3.1)	
False	943 (46.6)	435 (47.6)	157 (39.2)	351 (49.4)		575 (36.9)	368 (78.8)		107 (37.7)	830 (48.0)	
I don't know	1022 (50.5)	449 (49.2)	220 (54.9)	353 (49.6)	**<.0001**	934 (59.9)	88 (18.8)	**<.0001**	171 (60.2)	847 (49.0)	**0.002**
DRIA test											
True	49 (2.4)	26 (2.8)	17 (4.2)	6 (0.8)		41 (2.6)	8 (1.7)		9 (3.2)	38 (2.2)	
False	835 (41.2)	387 (42.4)	139 (34.7)	309 (43.5)		495 (31.8)	340 (72.8)		94 (33.1)	736 (42.5)	
I don't know	1141 (56.3)	500 (54.8)	245 (61.1)	396 (55.7)	**0.0003**	1022 (65.6)	119 (25.5)	**<.0001**	181 (63.7)	956 (55.3)	**0.01**
Allergy tests (e.g., prick test; IgE dosage, etc.)											
True	498 (24.6)	266 (29.1)	117 (29.2)	115 (16.2)		430 (27.6)	68 (14.6)		79 (27.8)	415 (24.0)	
False	956 (47.2)	436 (47.8)	152 (37.9)	368 (51.8)		594 (38.1)	362 (77.5)		109 (38.4)	841 (48.6)	
I don't know	571 (28.2)	211 (23.1)	132 (32.9)	228 (32.1)	**<.0001**	534 (34.3)	37 (7.9)	**<.0001**	96 (33.8)	474 (27.4)	**0.01**
Physician's diagnosis based on symptoms only											
True	249 (12.3)	130 (14.2)	85 (21.2)	34 (4.8)		173 (11.1)	76 (16.3)		40 (14.1)	204 (11.8)	
False	1516 (74.9)	653 (71.5)	258 (64.3)	605 (85.1)		1150 (73.8)	366 (78.4)		181 (63.7)	1329 (76.8)	
I don't know	260 (12.8)	130 (14.2)	58 (14.5)	72 (10.1)	**<.0001**	235 (15.1)	25 (5.4)	**<.0001**	63 (22.2)	197 (11.4)	**<.0001**
**MODULE 4 – MANAGEMENT**											
**The only treatment for LI is the exclusion of dairy products from the diet**											
True	802 (39.6)	319 (34.9)	136 (33.9)	347 (48.8)		612 (39.3)	190 (40.7)		91 (32.0)	707 (40.9)	
False	1081 (53.4)	508 (55.6)	235 (58.6)	338 (47.5)		807 (51.8)	274 (58.7)		155 (54.6)	920 (53.2)	
I don't know	142 (7.0)	86 (9.4)	30 (7.5)	26 (3.7)	**<.0001**	139 (8.9)	3 (0.6)	**<.0001**	38 (13.4)	103 (6.0)	**<.0001**
**A L-free diet may lead to vitamin D and calcium deficiency**											
True	819 (40.4)	337 (36.9)	165 (41.1)	317 (44.6)		639 (41.0)	180 (38.5)		104 (36.6)	714 (41.3)	
False	928 (45.8)	447 (49.0)	182 (45.4)	299 (42.1)		651 (41.8)	277 (59.3)		123 (43.3)	797 (46.1)	
I don't know	278 (13.7)	129 (14.1)	54 (13.5)	95 (13.4)	**0.04**	268 (17.2)	10 (2.1)	**<.0001**	57 (20.1)	219 (12.7)	**0.003**
**Cooking methods reduce the L content of foods**											
True	192 (9.5)	97 (10.6)	47 (11.7)	48 (6.8)		151 (9.7)	41 (8.8)		42 (14.8)	148 (8.6)	
False	1472 (72.7)	625 (68.5)	274 (68.3)	573 (80.6)		1086 (69.7)	386 (82.7)		173 (60.9)	1290 (74.6)	
I don't know	361 (17.8)	191 (20.9)	80 (20.0)	90 (12.7)	**<.0001**	321 (20.6)	40 (8.6)	**<.0001**	69 (24.3)	292 (16.9)	**<.0001**
**L in medications can trigger symptoms**											
True	1427 (70.5)	595 (65.2)	276 (68.8)	556 (78.2)		1082 (69.4)	345 (73.9)		179 (63.0)	1241 (71.7)	
False	255 (12.6)	124 (13.6)	54 (13.5)	77 (10.8)		178 (11.4)	77 (16.5)		40 (14.1)	214 (12.4)	
I don't know	343 (16.9)	194 (21.2)	71 (17.7)	78 (11.0)	**<.0001**	298 (19.1)	45 (9.6)	**<.0001**	65 (22.9)	275 (15.9)	**0.01**
**A L-free diet is beneficial/healthy even for those who are not lactose intolerant**											
True	362 (17.9)	124 (13.6)	99 (24.7)	139 (19.5)		338 (21.7)	24 (5.1)		58 (20.4)	302 (17.5)	
False	1306 (64.5)	641 (70.2)	234 (58.4)	431 (60.6)		873 (56.0)	433 (92.7)		158 (55.6)	1141 (66.0)	
I don't know	357 (17.6)	148 (16.2)	68 (17.0)	141 (19.8)	**<.0001**	347 (22.3)	10 (2.1)	**<.0001**	68 (23.9)	287 (16.6)	**0.002**
**L-free products are lower in calories than those containing lactose**											
True	131 (6.5)	65 (7.1)	26 (6.5)	40 (5.6)		119 (7.6)	12 (2.6)		32 (11.3)	98 (5.7)	
False	1622 (80.1)	714 (78.2)	326 (81.3)	582 (81.9)		1175 (75.4)	447 (95.7)		186 (65.5)	1427 (82.5)	
I don't know	272 (13.4)	134 (14.7)	49 (12.2)	89 (12.5)	0.40	264 (16.9)	8 (1.7)	**<.0001**	66 (23.2)	205 (11.8)	**<.0001**
**Eliminating dairy products from the diet can trigger LI**											
True	462 (22.8)	278 (30.4)	96 (23.9)	88 (12.4)		283 (18.2)	179 (38.3)		72 (25.4)	388 (22.4)	
False	1221 (60.3)	499 (54.7)	222 (55.4)	500 (70.3)		956 (61.4)	265 (56.7)		152 (53.5)	1061 (61.3)	
I don't know	342 (16.9)	136 (14.9)	83 (20.7)	123 (17.3)	**<.0001**	319 (20.5)	23 (4.9)	**<.0001**	60 (21.1)	281 (16.2)	**0.03**
**Eliminating L for a period may promote the onset of symptoms once reintroduced**											
True	850 (42.0)	463 (50.7)	173 (43.1)	214 (30.1)		533 (34.2)	317 (67.9)		109 (38.4)	737 (42.6)	
False	733 (36.2)	271 (29.7)	139 (34.7)	323 (45.4)		613 (39.3)	120 (25.7)		98 (34.5)	633 (36.6)	
I don't know	442 (21.8)	179 (19.6)	89 (22.2)	174 (24.5)	**<.0001**	412 (26.4)	30 (6.4)	**<.0001**	77 (27.1)	360 (20.8)	0.06
**There is a pharmacological treatment for LI**											
True	610 (30.1)	361 (39.5)	133 (33.2)	116 (16.3)		445 (28.6)	165 (35.3)		97 (34.2)	508 (29.4)	
False	1035 (51.1)	352 (38.6)	175 (43.6)	508 (71.4)		777 (49.9)	258 (55.2)		117 (41.2)	913 (52.8)	
I don't know	380 (18.8)	200 (21.9)	93 (23.2)	87 (12.2)	**<.0001**	336 (21.6)	44 (9.4)	**<.0001**	70 (24.6)	309 (17.9)	**0.001**
**Lactase supplements allow the digestion of L-containing food**											
True	1737 (85.8)	723 (79.2)	338 (84.3)	676 (95.1)		1281 (82.2)	456 (97.6)		213 (75.0)	1514 (87.5)	
False	42 (2.1)	25 (2.7)	6 (1.5)	11 (1.5)		36 (2.3)	6 (1.3)		8 (2.8)	34 (2.0)	
I don't know	246 (12.1)	165 (18.1)	57 (14.2)	24 (3.4)	**<.0001**	241 (15.5)	5 (1.1)	**<.0001**	63 (22.2)	182 (10.5)	**<.0001**

Abbreviations. LI: Lactose intolerance; L: Lactose.

11 respondents preferred not to specify their gender.
Correct answers presented in light green.
P-value based on Chi-square test.
